# Consultation and remediation in the north: meeting international commitments to safeguard health and well-being

**DOI:** 10.3402/ijch.v72i0.21231

**Published:** 2013-08-05

**Authors:** Laura Banfield, Cynthia G. Jardine

**Affiliations:** 1 Institute of Health Sciences, University of Oulu, Oulu, Finland; 2 Health Sciences Library, McMaster University, Hamilton, Ontario, Canada; 3 School of Public Health, University of Alberta, Edmonton, Alberta, Canada

**Keywords:** mining, remediation, contaminants, consultation, Giant Mine, community participation, Yellowknife, health, well-being

## Abstract

**Background:**

International commitments exist for the safeguarding of health and the prevention of ill health. One of the earliest commitments is the Declaration of Alma-Ata (1978), which provides 5 principles guiding primary health care: equity, community participation, health promotion, intersectoral collaboration and appropriate technology. These broadly applicable international commitments are premised on the World Health Organization's multifaceted definition of health. The environment is one sector in which these commitments to safeguarding health can be applied. Giant Mine, a contaminated former gold mine in the Northwest Territories, Canada, represents potential threats to all aspects of health. Strategies for managing such threats usually involve an obligation to engage the affected communities through consultation.

**Objective:**

To examine the remediation and consultation process associated with Giant Mine within the context of commitments to safeguard health and well-being through adapting and applying the principles of primary health care.

**Methods:**

Semi-structured interviews with purposively selected key informants representing government proponents and community members were conducted.

**Results:**

In reviewing themes which emerged from a series of interviews exploring the community consultation process for the remediation of Giant Mine, the principles guiding primary health were mapped to consultation in the North: (a) “equity” is the capacity to fairly and meaningfully participate in the consultation; (b) “community participation” is the right to engage in the process through reciprocal dialogue; (c) “health promotion” represents the need for continued information sharing towards awareness; (d) “intersectoral collaboration” signifies the importance of including all stakeholders; and (e) “appropriate technology” is the need to employ the best remediation actions relevant to the site and the community.

**Conclusions:**

Within the context of mining remediation, these principles form an appropriate framework for viewing consultation as a means of meeting international obligations to safeguard health.

International commitments exist regarding the promotion and protection of health and prevention of ill health for all peoples. Ranging from the World Health Organization's (WHO) definition of health in 1946, to the more recent report from the WHO Commission on the Social Determinants of Health (1), such documents are a call to all sectors to work towards achieving health for all.

The WHO defines health as “a state of complete physical, mental and social well-being and not merely the absence of disease or infirmity” (2). This definition presents a view of health that extends beyond the more commonly considered determinants of physical and mental health to include the entire life experience, indicating that responsibility for health does not lie solely within the health sector. The Lalonde Report, 1974, *New Perspectives on the Health of Canadians*, supports this through introducing the “Health Field Concept” as a tool for understanding and addressing health needs through 4 broad elements: human biology, environment, lifestyle and health care organization (3).

The Declaration of Alma-Ata, which resulted from the 1978 International Conference on Primary Health Care, “express[es] the need for urgent action by all governments, all health and development workers, and the world community to protect and promote the health of all the people of the world …” and lays out a set of principles towards achieving this goal (4,5). These principles include: equity, community participation, health promotion, intersectoral collaboration and appropriate technology. Equity refers to equal access to health services and health personnel for all members of the community (4–6). Community participation is the meaningful participation of the community in decision-making regarding the planning, implementation and maintenance of health services (4–6). Health promotion is “the concept of providing people with the information to control and improve their own health” (5). Intersectoral collaboration calls for action by sectors outside the provision of health services in coordinated efforts to support health (4–6). Appropriate technology is the implementation and adaptation of health services, interventions, technology and personnel in a manner suited to the needs and conditions of the community while remaining scientifically sound (4–6).

The principles of primary health care emphasize an approach in which the community has a strategic role. Primary health care was adopted by the WHO as the key to achieving “Health for All by 2000” (7). Although these principles emerge from a primary health care context, the Declaration of Alma-Ata remains a call to action that is broadly applicable. Indeed, Alma-Ata's principles can be applied as a framework within other areas including the environmental sector.

Subsequent charters and statements have responded to Alma-Ata's call to action by recognizing health and well-being as tied to social, economic and environmental factors. *Environment and Health, the European Charter and Commentary* of 1989 (8) integrates environment and health through principles, strategies and directives that incorporate intersectoral collaboration, community participation and shared decision-making. The 2005 *Bangkok Charter for Health Promotion in a Globalized World* (9) calls for civil society, corporations and government to be accountable to and adhere to regulations and international agreements that promote and protect health. Finally, the 2010 *Adelaide Statement on Health in All Policies* (10) emphasizes better health-centred policy at all levels and across all departments of government.

Together these documents demonstrate a history of the global recognition of the interplay between environment and health and the global commitment towards addressing concerns about health and well-being. More importantly, they are calls to action, providing principles that can be broadly applied in strategies towards mitigating health and well-being across sectors.

Using a case study of the consultation process for the remediation of a contaminated mining site in the Canadian North, Giant Mine, this paper demonstrates an application of the principles of primary health care from Alma-Ata—equity, community participation, health promotion, intersectoral collaboration and appropriate technology—within an intersectoral context. In so doing, this provides a framework for viewing consultation and remediation within the broader context of international commitments to safeguard health and well-being.

## Giant Mine

Giant Mine is a former gold mining operation situated on the outer boundaries of the City of Yellowknife, Northwest Territories, Canada (11). The mine is also adjacent to Great Slave Lake, the source of Canada's longest river, the Mackenzie River, with a river basin encompassing 20% of Canada's land mass and feeding into the Arctic Ocean (12). During operation from 1948 to 1999, over 7 million ounces of gold were removed from the mine and over 237,000 tonnes of arsenic trioxide were produced (13). Although surface contaminants are present, the main concern in the remediation of Giant Mine is the subsurface chambers containing the arsenic trioxide dust, believed to be the largest containment of this toxic substance in the world (14). Arsenic is both colourless and tasteless, and easily dissolvable in water (15,16). Initially, the arsenic trioxide dust was stored in chambers below the permafrost, which acted to freeze it *in situ*. Later, it was stored in chambers within the permafrost layer; a 1977 report by the Canadian Public Health Association, deemed this an acceptable solution (17). However, thawing of the permafrost has allowed seepage of water into the chambers. This water is presently being collected and treated at a facility onsite before being released back into the environment (18).

Giant Mine has a complex history characterized by concerns over the impact of arsenic contamination in the air and water and a challenging labour history. Though levels of arsenic released directly into the environment declined over time, accounts of deaths of animals and children that were attributed to arsenic contaminated snow and melt-water remain part of the community's experience with the Mine (19–21). Concerns also remain over the issues of adequate compensation for the loss of land and for the discovery of the mine (19–21). In 1992–93, there was an 18 month miners’ strike/lockout. During the strike, an explosion at the mine killed 9 workers; 1 man was later charged and convicted. Media reports have described this as one of the most deadly and volatile strikes in Canadian labour history (22,23).

Royal Oaks Mines Inc., then owner of the mine, entered receivership in 1999 and responsibility for Giant Mine was transferred to Indian and Northern Affairs Canada (INAC), presently referred to as Aboriginal Affairs and Northern Development Canada (AANDC) (24). Under the Commissioner's Lands Act, 1970, surface land transfers, including the area encompassing Giant Mine, were made to the Government of the Northwest Territories (GNWT) (24). Thus INAC and GNWT became responsible for the subsurface and surface aspects of Giant Mine, including the contaminants.

From 2001 to 2005, Miramar Giant Mine Ltd took over the assets of the mine and continued operation, but were not held responsible for pre-existing environmental liabilities. Miramar submitted an abandonment and restoration plan in 2001 (24).

In 2001, the first report exploring arsenic management options, *Study of Management Alternatives – Giant Mine Arsenic Trioxide Dust*, was released and intentional engagement with the public occurred through technical workshops (24). The 2003 final report, *Arsenic Trioxide Management Alternatives – Giant Mine*, and more consultation through public workshops followed this. By 2004, a decision was made to proceed with the frozen block method^1^ and by 2005, the mine was officially designated as abandoned (24).

Also in 2005, INAC and GNWT signed a co-operation agreement for the management and remediation of the Mine, thereby becoming the project co-proponents. In a news release, it was stated that the “agreement will allow us to effectively deal with the toxic inheritance left by the Giant Mine and to protect human health and ensure public safety” (25). While the reference to “toxic inheritance” likely refers to the arsenic and other contaminants, it could be argued that the history of the site has also left a further toxic legacy that must be addressed.

The Giant Mine Remediation Plan was developed and submitted to the Mackenzie Valley Land and Water Board (MVLWB) in application for a water license to proceed with remediation in 2007. Due to concerns raised by the community, the plan was eventually referred to the Mackenzie Valley Environmental Impact Review Board (MVEIRB) for environmental assessment (EA) in 2009. The terms of reference for the EA clearly stated the purpose of public consultation as being “to provide those individuals who may be affected by the development an opportunity to effectively participate in the environmental assessment” (27). Further rounds of consultation and additional documents from both the government proponents and the community were exchanged and submitted for consideration to the MVEIRB. Closing comments in the EA were submitted as of October 2012.

### Consultation

Within the context of resource development and remediation activities in Canada, a number of acts, regulations and guidelines mandated a duty to consult with the affected community or communities. Two of the acts relevant to this project are the Mackenzie Valley Resource Management Act and the Canadian Constitution Act, 1982. The Mackenzie Valley Resources Management Act indicates who must be contacted, and when, for consultation around various uses of the land (28). Within the Canadian Constitution Act, section 35 protects the rights of Aboriginal peoples and treaties (29). Commonly known as the “duty to consult”, in this instance it was operationalized around the need to consult with Aboriginal peoples regarding proposed activities affecting the land (30). Specific to remediation, AANDC provides a *Mine Site Reclamation Policy for the NWT* and accompanying guidelines (31,32). One of the core objectives is to minimize the impact of mining activities on the environment and human health and safety.

## Methods

A constructivist grounded theory approach was used to examine the effectiveness of community consultation information in the development of the Giant Mine Remediation Plan (33). This approach was used because it is a systematic, flexible and comparative methodology for inductive qualitative research. The interviews from this study are also being used for a larger project exploring risk communication activities and public trust.

Key informants were purposively selected and collectively represented community members as well as government proponents. Those classed as “community members” included representatives from the City of Yellowknife, Yellowknives Dene First Nation, Giant Mine Community Alliance, Alternatives North, and the Native Women's Association of the Northwest Territories. All identified parties that were contacted, with the exception of one, agreed to a recorded interview – no representative from the North Slave Metis Alliance was available during the 2 interview periods. The key informants from the government proponents represented AANDC (then INAC) and GNWT.

Semi-structured interviews, incorporating questions regarding engagement in and effectiveness of the consultation process were conducted between November 2010 and February 2011. The interviews were then transcribed and inductively coded for themes characterizing the consultation process through applying a constant-comparative and concept-development approach (34). Data validation occurred through member checking, with each interviewee being provided an opportunity to review their full transcript and coded sections within the identified themes (35).

Ethics approval for this research was obtained from the University of Alberta Health Research Ethics Board. A Northwest Territories Scientific Research Licence was obtained through the Aurora Research Institute.

## Results

In reviewing themes that emerged from the interviews on the consultation process for the remediation of Giant Mine, connections to the principles of primary health care as outlined in Alma-Ata were identified. With some adaptation to the descriptions, these principles form the framework for analyzing the consultation process within the context of international commitments to safeguard health. This interpretation is described accompanied by selected comments from the interviewees. For the purposes of preserving anonymity, interviewees are identified as either “community member” or “government proponent”.

### Equity

Equity refers to equal access to health services and health personnel for all members of the community (5). Within the context of consultation and Giant Mine, equity becomes the capacity to fairly and meaningfully participate in consultation through the provision of information, and the ability to engage at the same level across all stakeholders. For the community members, it means access to consultants and advisors who can help the community understand the technical details, safety concerns, health risks and magnitude of the project. As one community member indicated, “Where it gets complex is where it extends to under-asseted, under-funded, under-staffed groups, people who are included in the consultation … [T]here's people that can manage it and then there's other people that are challenged by it”. Another respondent reinforces this:

[T]hey've had a lot of workshops, open houses, but they haven't given anybody the real tools to properly engage in this . … [I]t takes money to review this stuff and capacity, but where does capacity come from? It comes from money and being able to retain your own independent technical experts and having the luxury of some time and resources to help you work your way through that stuff. And that has been lacking throughout this process.

(Community Member)

### Community participation

Community participation is the meaningful participation of the community in decision-making regarding the planning, implementation and maintenance of health services (5). Applying this to consultation and Giant Mine, community participation is the right to participate in decision-making regarding proposed remediation activities through reciprocal dialogue with the project proponents. Reciprocal dialogue can be challenging due to the varying perspectives and interests of those involved. However, it is the preferred means to explore the concerns of all parties and, through discussion, to negotiate and prioritize the means of addressing those concerns. One community member described their ideal consultation as an exchange between the parties involved in which an opportunity exists to be heard and to see one's self in the decision, but questions the motivation and the disconnect in the decisions made in the case of Giant Mine where AANDC holds the decision-making power.

I do believe that there are people in Yellowknife that are trying to do the right thing. In Ottawa I'm not convinced of that. And certainly higher up in Ottawa, that's not happening. I think it depends on where within DIAND [AANDC]. I think for most of DIAND [AANDC], the objective here is to try to—they'll say this—protect the environment and human health, and those are basic things. And I think that's what they're trying to do, but they're being driven by cost and whatever.

(Community Member)

### Health promotion

Health promotion involves “providing people with the information to control and improve their own health” (5). Within the context of the remediation of Giant Mine, health promotion translates to building a broad and continuing awareness within the community about what is happening at the mine, the impact on the environment and how it influences health and well-being towards enabling the community to more effectively participate in consultation, decision-making and long-term monitoring. To achieve this, there is a need for continued information sharing and reporting on site monitoring, as well as education. As one participant noted, the goal should be to keep communication about Giant Mine in the forefront so that people no longer have questions about what is happening at the site:

It's giving them the information that will hopefully give them a comfort level that, okay, this is going to be a safe site. The stuff's still there, it's not going anywhere, but it's not going to get into the water, it's not going to get into the air, the animals aren't going to have access to it, it's not going to affect people.

(Government Proponent)

### Intersectoral collaboration

Intersectoral collaboration calls for action by sectors outside health services in a coordinated effort to support health (5). Applying this to the case of Giant Mine, intersectoral collaboration is the inclusion of all stakeholders towards addressing the multifaceted concerns raised by the community during the process of consultation. In doing so, the challenges to consultation presented by legacy concerns, such as compensation and past events that are outside the responsibility of the proponent, may be given a forum in which they can be discussed and possibly addressed. Involving other departments and parties may lead to a remediation solution and consultation process that takes into account the whole impact of the site, environmental, historical, economic and social, on the community. The need for recognizing these legacy issues is evident in the following comments:

And I understand how challenging that must be for INAC … You're being blamed for something that you think you didn't do, but you can't ignore that because it is a reality in the First Nation. And so the fact—and so all the dogs dying, and like the big thing that nobody has really recognized for me, and no one's going to go back and talk about these issues …. Every time they meet with the community, the community talks about compensation for what's happened.

(Community Member)

### Appropriate technology

Appropriate technology is the implementation of health services, interventions, technologies and personnel in a manner adapted to the needs and conditions of the community (5). As applied to consultation and Giant Mine, appropriate technology is the use of the best remediation and consultation activities relevant to the site and the community. This includes the technical requirements to implement, maintain and monitor the remediation activities as well as the communication and consultation strategies used and the people responsible for executing them. Descriptions emerged from the interviews detailing appropriate strategies for engaging in consultation with the various community groups—using models and plain language, visiting the mine and meeting with the Band Chiefs and Council as a starting point. This was described by one respondent when commenting on how consultation has been carried out:

I think that you need to get the communications people actually out of it, and have local people that have actually interacted with the First Nations, because I would say that was one of the big barriers for the Giant staff at first, is in terms of their sort of regulatory approach to community engagement. They had nobody that really understood how the communities worked and what they needed to do. And I think that's changed some and continues to change, and that's good, and it's pretty important, connecting, because that's what you're trying to do. You're trying to create a relationship, and if you're going to work there for 15, 25 years, it's not just about today and what happened in the past. You have got to overcome 50 years of history, too. And so, it very much has to be a long-term approach and you've got to have people that are going to be there for a long time, and the folks that are going to come to these things will see the same face.

(Community Member)

## Conclusion

In viewing the remediation and community consultation activities associated with Giant Mine within the context of international commitments to preserve and safeguard health and well-being, such activities become part of answering the broader calls to action on “health for all” contained within the Declaration of Alma-Ata, the Adelaide Statement, Bangkok Charter and European Charter through intersectoral collaboration and recognition of environmental impacts on health.

As noted earlier, the WHO defines health as a “complete state of physical, mental, and social health and well-being” (2). There is a clear connection between the remediation of Giant Mine and the entire spectrum of health as evidenced by the risk to physical health represented by the arsenic contamination and to well-being as evidenced by the lasting impact of the history of the site on the community. Through the Giant Mine Remediation Plan, action is being taken towards mitigating the potential effects of arsenic and other site contaminants on the environment and consequently human health. However, respondents’ comments suggest there are unresolved legacy concerns related to past practices and events, and compensation, affecting the well-being of the community, which are not being addressed.

Using Giant Mine as an example, by applying and modifying the principles of primary health care from the Declaration of Alma-Ata, a framework for developing a set of consultation and remediation principles emerges. Such a framework has the potential to address the complex issues affecting health and well-being raised during the consultation process through developing community-centred strategies based on equity and capacity, community participation in shared decision-making, recognition of health promotion as awareness of risk, intersectoral collaboration as engaging all stakeholders, and the use of community appropriate technologies and strategies.

As long as there are resource development activities in the North, there will be a need to engage in community consultation towards remediation strategies that address the varied detrimental effects of mining. It is hoped that industrial processes and government policies will continue to develop and inform improved industry practices that lessen the impact of mining on the environment and surrounding communities. To whatever extent this is achievable, community consultation will remain a critical piece of the remediation process. It is in the best interest of all parties to engage in a consultation process grounded in a set of mutually agreed upon principles that prioritize the health and well-being of the community.

## Footnotes

1 The frozen block method refers to a process by which the arsenic chambers and the rock around them are frozen in a cup-shaped block. The frozen state is maintained using passive thermosyphons designed to draw heat away from the block (26). If the system were to fail, it would take decades for the blocks to thaw (13).

## References

[1] Commission on the Social Determinants of Health (2008). Closing the gap in a generation: health equity through action on the social determinants of health: final report of the commission on social determinants of health.

[2] World Health Organization. http://www.who.int/about/definition/en/print.html.

[3] Lalonde MA (1974). New perspective on the health of Canadians: a working document.

[4] World Health Organization (1978). Declaration of Alma-Ata. Primary health care: report of the International Conference on Primary Health Care.

[5] Seear M (2012). Introduction to international health.

[6] College & Association of Registered Nurses of Alberta (CARNA) (2005). Primary health care.

[7] World Health Organization (1981). Global strategy for health for all by the year 2000.

[8] World Health Organization – Europe (1989). Environment and health: the European charter and commentary.

[9] Bangkok Charter (2005). The Bangkok Charter for Health Promotion in a Globalized World. http://www.who.int/healthpromotion/conferences/6gchp/bangkok_charter/en/.

[10] World Health Organization (2010). Adelaide statement on health in all policies. http://www.who.int/social_determinants/hiap_statement_who_sa_final.pdf.

[11] Aboriginal Affairs and Northern Development Canada Giant Mine Remediation Project. http://www.aadnc-aandc.gc.ca/eng/1100100027364/1100100027365.

[12] Grant J, Dagg J, Dyer S, Lemphers N (2010). Northern lifeblood: empowering northern leaders to protect the Mackenzie River Basin from oil sands risks.

[13] Aboriginal Affairs and Northern Development Canada The giant story. http://www.aadnc-aandc.gc.ca/eng/1100100023215/1100100023227.

[14] Aboriginal Affairs and Northern Development Canada How much is 237 000 tonnes of arsenic trioxide dust?. http://www.aadnc-aandc.gc.ca/eng/1100100027425/1100100027429.

[15] Health Canada Arsenic in drinking water. http://www.hc-sc.gc.ca/hl-vs/iyh-vsv/environ/arsenic-eng.php.

[16] McGuigan CF, Hamula CLA, Huang S, Gabos S, Le XC (2010). A review on arsenic concentrations in Canadian drinking water. Environ Rev.

[17] CPHA (Canadian Public Health Association) (1977). Task force on arsenic. Final Report. Canadian Public Health Association, Yellowknife, Northwest Territories. As cited by Aboriginal Affairs and Northern Development Canada. Arsenic Trioxide at Giant Mine.

[18] Aboriginal Affairs and Northern Development Canada Arsenic trioxide at Giant Mine. http://www.aadnc-aandc.gc.ca/eng/1100100027422/1100100027423.

[19] Sandlos J, Keeling A Giant Mine: historical summary. Submission to Giant Mine environmental assessment.

[20] Mackenzie Valley Environmental Impact Review Board (2008). Giant Mine remediation plan, proposed by INAC Contaminants & Remediation Directorate: scoping hearing, day 2 of 2 (transcript). Explorer Hotel, Yellowknife NWT Canada.

[21] Yellowknives Dene First Nation (2008). Presentation to MVEIRB issues scoping session, EA0809-001 Giant Mine Remediation. http://www.reviewboard.ca/upload/project_document/EA0809-001_Presentation_from_YKDFN_1328900961.pdf.

[22] Giant Mine legacy still haunts city, says former councillor (2012). CBC News North. http://www.cbc.ca/news/canada/north/story/2012/09/18/north-giant-mine-20th-anniversary.html.

[23] Laidlaw K (2012). The murders in the mine. Up here.

[24] Aboriginal Affairs and Northern Development Canada Historical timeline – Giant Mine Remediation Project. http://www.aadnc-aandc.gc.ca/eng/1100100023233/1100100023235.

[25] Aboriginal Affairs and Northern Development Canada Canada, GNWT Agree to Cooperate on Giant Mine.

[26] Aboriginal Affairs and Northern Development Canada Frozen Block Method – Giant Mine Remediation Project. http://www.aadnc-aandc.gc.ca/eng/1100100027419/1100100027420.

[27] Mackenzie Valley Environmental Impact Review Board Terms of reference for the environmental assessment of the Indian and Northern affairs Canada Giant Mine remediation. http://www.reviewboard.ca/upload/project_document/1244589258_Terms_of_Reference-_Giant_EA.PDF.

[28] Government of Canada Mackenzie Valley Resource Management Act, S.C. 1998, c. 25. http://laws-lois.justice.gc.ca/eng/acts/M-0.2/index.html.

[29] Government of Canada Constitution Act, 1982. C.11(UK) Schedule B, Part 2, Section 35. Rights of the Aboriginal Peoples of Canada.

[30] Aboriginal Affairs and Northern Development Canada (2011). Aboriginal consultation and accommodation – updated guidelines for federal officials to fulfill the duty to consult. http://www.aadnc-aandc.gc.ca/eng/1100100014664/1100100014675.

[31] INAC - Aboriginal Affairs and Northern Development Canada (2002). Mine site reclamation policy for the Northwest Territories. http://www.aadnc-aandc.gc.ca/eng/1100100036038/1100100036040.

[32] INAC – Aboriginal Affairs and Northern Development Canada – Renewable Resources and Environment Mine site reclamation guidelines for the Northwest Territories. http://www.aadnc-aandc.gc.ca/eng/1100100024558/1100100024569.

[33] Charmaz K (2006). Constructing grounded theory: a practical guide through qualitative analysis.

[34] Strauss AL, Corbin J, Denzin NK, Lincoln YS (1998). Grounded theory methodology: an overview. Strategies of qualitative inquiry.

[35] Lincoln YS, Guba EG (1985). Naturalistic inquiry.

